# MicroRNA-296-5p is differentially expressed in individuals with and without HIV-1 infection

**DOI:** 10.1590/1678-4685-GMB-2020-0017

**Published:** 2020-06-22

**Authors:** Jhonathan Cárdenas-Bedoya, Jazmin Marquez-Pedroza, María Cristina Morán-Moguel, Martha Escoto-Delgadillo, Eduardo Vázquez-Valls, Gracia Viviana González-Enríquez, Alma Minerva Pérez-Ríos, Blanca Miriam Torres-Mendoza

**Affiliations:** 1 Instituto Mexicano del Seguro Social, Centro de Investigación Biomédica de Occidente, Laboratorio de Inmunodeficiencias y Retrovirus Humanos, Guadalajara, Jalisco, Mexico.; 2 Universidad de Guadalajara, Departamento de Biología Molecular y Genómica, Guadalajara, Jalisco, Mexico.; 3 Universidad de Guadalajara, Departamento de Fisiología, Guadalajara, Jalisco, Mexico.; 4 Universidad de Guadalajara, Centro Universitario de Ciencias de la Salud, Departamento de Disciplinas Filosófico, Metodológicas e Instrumentales, Guadalajara, Jalisco, Mexico.; 5 Universidad de Guadalajara, Centro Universitario de Ciencias Biológicas y Agropecuarias, Guadalajara, Jalisco, Mexico.; 6 Secretaría de Salud, Dirección de Generación de Recursos Profesionales, Investigación y Desarrollo, Jalisco, Mexico.; 7 Instituto Mexicano del Seguro Social, Hospital Regional 110, Guadalajara, Jalisco, Mexico.

**Keywords:** miR-296-5p, HIV-1, naïve, PIN1

## Abstract

MicroRNAs are considered as potential biomarkers, agents, or therapeutic targets; few studies have addressed the expression of miRNAs in treatment-naïve patients infected with HIV-1. The aim of this study was to assess plasma relative circulating miRNA expression profiles in treatment-naïve Mexican patients with HIV/AIDS and healthy individuals using a commercial array. A low CD4+ T cell count and high viral load were found in all patients. Decreased relative miRNA-296-5p expression was observed in patients; moreover, this was the only miRNA that showed differences between the two groups. Thus, we measured the absolute expression of miR-296-5p by qPCR, confirming the result with statistically significant differences (P < 0.05). There is evidence that miR-296-5p regulates the expression of the *PIN1* gene, which encodes the peptidylprolyl Cis/Trans isomerase NIMA-Interacting-1, that is involved in different stages of the biological cycle of HIV-1, this relationship is corroborated by bioinformatics analysis and ELISA assay was used to measure plasma levels of PIN1. The decreased expression of miR-296-5p found in naïve patients with HIV infection suggests a regulatory activity of this miRNA on virus replication, making it a potential therapeutic agent against HIV. Finally, miR-296-5p could be inhibiting the virus transcription by regulating genes different than *PIN1*.

Acquired immunodeficiency syndrome (AIDS) has no cure; however, it can be controlled by antiretroviral therapy (UNAIDS, 2018). The search for new therapies that control the virus led to new research lines, e.g., microRNAs (miRNAs), which are chains of noncoding RNAs of 20–25 nucleotides that regulate gene expression by binding to complementary bases on specific sites of mRNAs, thus inhibiting their translation or degrading them (Allantaz *et al.*, 2012).

In HIV/AIDS pathogenesis, some cellular miRNAs have an antiviral action during the infection and replication of the virus. The identification of miRNA expression differences between patients with HIV treatment-naïve or healthy conditions will clarify the mechanisms of regulation of viral replication (Swaminathan *et al.*, 2014; Liao *et al.*, 2017). Few studies have addressed miRNA expression in HIV-infected patients in their basal state (without antiretroviral treatment). The aim of this study was to assess plasma relative circulating miRNA expression profiles in treatment-naïve Mexican patients with HIV/AIDS and healthy individuals.

This study was approved by the Ethics and Research Committee of the participating institution (R-2014-1305-11). Written informed consent was obtained from all subjects.

Thirteen male individuals were divided into two groups, as follows. Group 1: 10 treatment-naïve HIV-1-positive patients who were being followed at the Laboratorio de Inmunodeficiencias y Retrovirus Humanos, Centro Médico Nacional de Occidente of the Instituto Mexicano del Seguro Social; and Group 2 (control): three voluntary individuals without HIV-1 infection. Subsequently, for the absolute expression, 10 new volunteers without HIV infection were selected (Group 3).

Because of the small sample size, the standardization criteria were strictly fulfilled by increasing the internal validity through the homogenization of the groups, using strict internal controls, and applying normalization processes.

Individuals with hepatitis B or C, influenza, tuberculosis, diabetes, cardiovascular disease, or cancer were excluded from the study. All participants were male because it has been shown that the hormonal changes that occur in women can modify miRNA expression (Klinge, 2015; Chen *et al.*, 2016; Rao *et al.*, 2016).

Clinical and demographic data were collected for HIV-1-positive patients, CD4^+^ and CD8^+^ cells were quantified by flow cytometry (Cytomics FC500, Beckman Coulter), and viral load was determined using an Arthus® HI Virus QS-RGQ kit on a QIAsymphony® SP/AS sample extraction and preparation apparatus, and a Rotor-Gene Q® real-time PCR machine.

Total RNA was isolated from plasma using a miRNeasy Serum/Plasma kit (QIAGEN, Hilden, Germany), according to the manufacturer's instructions. The miRNA isolation efficiency was controlled based on the recovered amount of the *Caenorhabditis elegans* miR-39 added during the extraction. The RNA concentration was evaluated by spectrophotometry using NanoDrop 2000/2000c (Thermo Fisher Scientific, Waltham, MA, USA); the purity of the RNA was obtained based on the A260/A280 ratio. RNA integrity was evaluated by electrophoresis on 1.5% agarose gels with formaldehyde, and RNA samples were stored at −80°C until use.

A miScript II RT kit was used for cDNA synthesis and miScript miRNA PCR Array and miScript SYBR® Green PCR kits (QIAGEN) were used to analyze the relative expression of the 84 miRNAs that were most relevant to pathophysiological conditions and were detectable and differentially expressed in serum, plasma, and other bodily fluids. Quantitative real-time PCR (qPCR) conditions were as per the manufacturer's instructions and a Rotor-Gene Q® instrument was used to perform this experiment.

The expression of miRNAs was analyzed using the miScript miRNA PCR Array Data Analysis Tool (QIAGEN) (http://pcrdataanalysis.sabiosciences.com/mirna/arrayanalysis.php). The relative miRNA expression levels were calculated using the Cq comparative method. Changes in the expression levels of miRNAs were calculated using the 2^–DDCq^ equation (Fold Change). Mann-Whitney U-test (two-tailed *P* values) was used to examine the differential expression of miRNAs between groups. Significance was set at *P* < 0.05. Expression data were presented as means ± standard error of the mean (SEM).

The expression levels were normalized using the stable miRNAs identified in the array (miR-200b-3p, miR-92a-3p, miR-193a-5p, and miR-103a-3p). The analysis was performed by combining the GenEx version 6 (http://www.biomcc.com/genex-software.html) and RefFinder (http://leonxie.esy.es/RefFinder/) algorithms which evaluate the relative expression to identify the best internal references (Vandesompele *et al.*, 2002; Marabita *et al.*, 2016).

The clinical and demographic data were as follows. Group 1: age, 30.7 ± 8.4 years; CD4+ Cells, 181.4 ± 157.3 and CD8+, 947.63 ± 690.8 cells in blood (numbers of cells per microliter); viral load, 1,703,873 ± 3,330,887 copies of HIV per milliliter; Group 2: age, 36.3 ± 9.3 years. The time elapsed since diagnosis was 0–5 months in nine patients and 8 years in one patient. Four of ten patients in Group 1 were classified as stage 2 and six of them as stage 3, according to the Centers for Disease Control and Prevention guidelines (CDC, 2018).

Of the 84 miRNAs evaluated in the array, only miR-296-5p was significantly underexpressed in group 1 (0.093 ± 0.033; *P* =0.0225) compared with the group 2 (control group healthy) (1.000 ± 0.298) (Figure 1). Therefore, the absolute expression of miR-296-5p was analyzed by qPCR in all individuals in group 1 and 3. The assays were performed in duplicate for each sample using a standard curve, in which a serial dilution of enriched synthetic miR-296-5p (10^9^–10^3^ copies) was performed. Group 1 exhibited 2.28 10^10^ ± 3.16 10^10^ copies of miR-296-5p, and the Group 3 had 2.62 10^11^ ± 1.34 × 10^11^ copies of miR-296-5p (*P* < 0.05).

**Figure 1:**
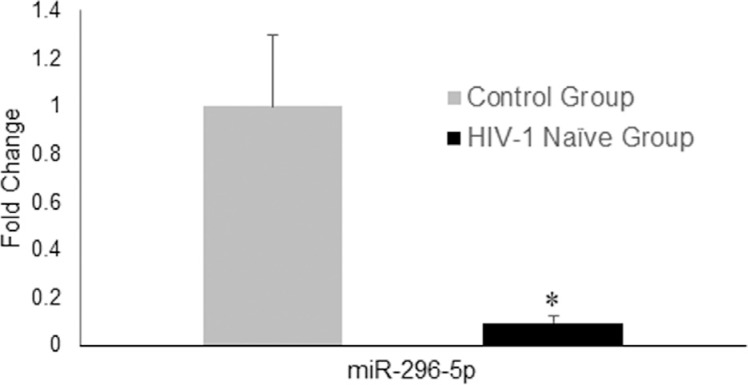
MicroRNA-296-5p relative expression in plasma from healthy individuals and treatment-naïve HIV-1-positive patients. The expression was normalized with miR-200b-3p, miR-92a-3p, miR-193a-5p, and miR-103a-3p. * Indicates statistically significant differences (*P* < 0.05) by Mann-Whitney U-test.

For the identification of the miR-296-5p target genes, the public database miRTarBase was accessed because it gathers functional studies of miRNA – target interactions, which are validated experimentally (http://miRTarBase.cuhk.edu.cn/) (Chou *et al.*, 2018; Huang *et al.*, 2020); at least 10 genes showed strong evidence of interaction with miR-296-5p, of which peptidylprolil cis / trans isomerase, the gene that interacts with NIMA 1 (*PIN1*), is the only one involved in three different points of the biological cycle of the virus (disassembly or denaturation of the viral capsid, the reverse transcription of the viral RNA and the integration of the HIV-1 cDNA into the host genome). The program miRanda-mirSVR (http://www.microrna.org/microrna/releaseNotes.do) was used for the confirmation of binding sites to these genes (Table 1). Three miR-296-5p-binding sites of the *PIN1* gen (NM_006221) in the 129nt, 146nt and 413nt positions were found.

**Table 1:** miR-296-5p/*PIN1* Alignment.

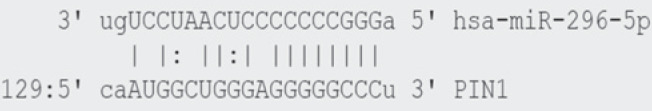	mirSVR score: -0.1209*
	PhastCons score: 0.5452*
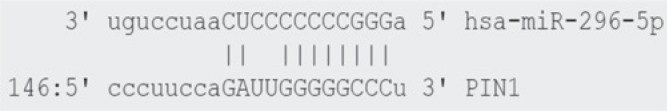	mirSVR score: -0.1136*
	PhastCons score: 0.5452*
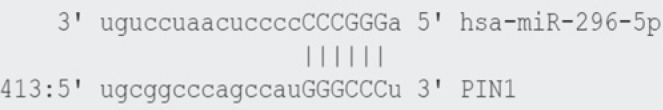	mirSVR score: -0.0024*
	PhastCons score: 0.5525*

* Restricted analysis “View targets sites of conserved miRNAs with good mirSVR score” (< =-0.1). And PhastCons score (> =0).

*PIN1* is also involved in different stages of the biological cycle of HIV-1: Misumi *et al.* (2010) determined that the alteration or restriction in PIN1 expression generates an increase in the number of viral capsids within the cytoplasm of T lymphocytes, leading to the restriction of HIV-1 infection; Watashi *et al.* (2008) found that PIN1 modulates the expression of apolipoprotein B mRNA-editing enzyme, polypeptide-like 3G catalytic (APOBEC3G), which restricts the replication of HIV-1 by interfering in the reverse transcription process, and Manganaro *et al.* (2010) determined that PIN1 catalyzes a conformational modification of the HIV-1 integrase that is required to increase its stability, which is necessary for an effective viral infection.

In this study, the expression of miRNAs in plasma of treatment-naïve HIV-infected patients from Western Mexico was determined. The miR-296-5p was the only one differentially expressed. The participation of this miRNA has been essentially related in studies of cancer patients (Lee *et al.*, 2014; Shi *et al.*, 2018; Wang *et al.*, 2019; Zhou *et al.*, 2019). However, in viral infections merely three studies have evaluated its expression levels. Two of them analyzed the efficiency of miR-296-5p regulation over EV71 (Enterovirus 71) and IAV (influenza A virus) *in vitro* showing this miRNA regulates viral replication of both viruses (Zheng *et al.*, 2013; Gao *et al.*, 2019). The third trial was conducted on HIV, however this miRNA was not discussed by the author, nor was the regulatory capacity of the miRNA on the virus demonstrated, Bignami *et al.* (2012) compared a group of treatment-naïve HIV-infected patients against a group of individuals exposed to the virus but not infected. They found a lower expression levels of miR-296-5p in treatment-naïve patients HIV-infected in comparison with the other group; this outcome is similar to our results where the treatment-naïve patients HIV infected showed a lower expression levels of miR-296-5p towards the group of without infection individuals. However, it has not been determined which is the target of regulation of this miRNA in HIV.

Due to the regulatory activity associated to miR-296-5p over *PIN1* (Lee *et al.*, 2014), which is given at post-transcriptional level, the quantification of the PIN1 protein was performed in triplicated in both groups using ELISA method (MyBioSource); the findings resulted without any significative difference (calculated by the two-tailed Mann-Whitney U-test; *P* = 0.1177) between Group 1 and Group 3, with values of 111.71 ± 81.35 U/L and 74.32 ± 22.77 U/L respectively. It is necessary to emphasize that our assay was carried out in treatment-naïve patients HIV-infected plasma samples (*in-vivo*) unlike the only previous study in which these two genes are related using cell cultures (Lee *et al.*, 2014). In the other hand, we have to consider that a miRNA can have multiple target genes and a gen can have multiple regulatory miRNAs such as *PIN1*, which not only is regulated by miR-296-5p but also there are others miRNAs responsible of its regulation like miR-140-5p (Yan *et al.*, 2017) and miR-874-3p (Leong *et al.*, 2017).

The effectiveness of miRNA-mediated regulation of a gen is based on the base-paring mechanism between the complementary sequences of miRNA and the mRNA target (Wang *et al.*, 2019), therefore the suppression or degradation of mRNAs depends on this complementary. A mutation or polymorphism in the seed sequences of these RNAs could lead to alterations in their specificity towards a gen. Using bioinformatic prediction tools, it has been revealed that a single nucleotide polymorphisms (SNPs) in the seed regions of a mature miRNA can change the number of target genes and generate new targets. Along with the miRNAs, the proteins variations in the regulatory or coding sequences of their genes can modulate their intra and extracellular concentration. An example is the −842 G/C variant (rs2233678) in the promotor of *PIN1* gen, in which individuals with −842G allele increase the transcriptional effectiveness of *PIN1*, thus the expression of PIN1 protein. On the contrary, individuals having the −842C allele showed a reduced transcriptional effectiveness of *PIN1* and a lower PIN1 protein concentration in serum (Hou *et al.*, 2015). Although the aim of this study was not determined polymorphisms of *PIN1*, we assume that this could be the reason of the lack of differences between the concentrations of PIN1 among groups. However, it is necessary to perform subsequent studies that allow the identification of the variants and not only the quantification of circulating levels of the protein but also its intracellular concentration.

This is the first study that confirms the subexpression of miR-296-5p in treatment-naïve HIV-positive patients compared with healthy individuals, suggesting a regulatory activity of this miRNA on virus replication, making it a potential therapeutic agent against HIV. In addition, the discordance on the expression of the PIN1 protein among evaluated groups, proposes that miR-296-5p could be inhibiting the viral transcription by the regulation of other genes different to *PIN1*.
